# Integrin α_v_β_3_-targeted polydopamine-coated gold nanostars for photothermal ablation therapy of hepatocellular carcinoma

**DOI:** 10.1093/rb/rbab046

**Published:** 2021-08-10

**Authors:** Yang Li, Ping Hu, Xiali Wang, Xu Hou, Fengzhen Liu, Xiaohong Jiang

**Affiliations:** 1 Zhong Yuan Academy of Biological Medicine, Liaocheng People’s Hospital, No. 67 Dongchang West Road, Liaocheng 252000, China; 2 Clinical Laboratory, Liaocheng People’s Hospital, No. 67 Dongchang West Road, Liaocheng 252000, China; 3 Department of Hepatobiliary Surgery, Liaocheng People’s Hospital, No. 67 Dongchang West Road, Liaocheng 252000, China; 4 Liaocheng People’s Hospital, Medical College of Liaocheng University, No. 67 Dongchang West Road, Liaocheng 252000, China; 5 School of Basic Medical Sciences, Shandong University, No.44 Wenhua West Road, Jinan 250012, China

**Keywords:** polydopamine-coated gold nanostars, RGD peptide, targeted photothermal therapy, hepatocellular carcinoma

## Abstract

Photothermal therapy (PTT) has emerged as a promising cancer therapeutic method. In this study, Arg-Gly-Asp (RGD) peptide-conjugated polydopamine-coated gold nanostars (Au@PDA-RGD NPs) were prepared for targeting PTT of hepatocellular carcinoma (HCC). A polydopamine (PDA) shell was coated on the surface of gold nanostars by the oxidative self-polymerization of dopamine (termed as Au@PDA NPs). Au@PDA NPs were further functionalized with polyethylene glycol and RGD peptide to improve biocompatibility as well as selectivity toward the HCC cells. Au@PDA-RGD NPs showed an intense absorption at 822 nm, which makes them suitable for near-infrared-excited PTT. Our results indicated that the Au@PDA-RGD NPs were effective for the PTT therapy of the α_V_β_3_ integrin receptor-overexpressed HepG2 cells *in vitro*. Further antitumor mechanism studies showed that the Au@PDA-RGD NPs-based PTT induced human liver cancer cells death via the mitochondrial–lysosomal and autophagy pathways. *In vivo* experiments showed that Au@PDA-RGD NPs had excellent tumor treatment efficiency and negligible side effects. Thus, our study showed that Au@PDA-RGD NPs could offer an excellent nanoplatform for PTT of HCC.

## Introduction

Hepatocellular carcinoma (HCC) is a common and lethal primary liver malignancy, which has increased morbidity and mortality worldwide [1]. Surgical resection, systemic chemotherapies, liver transplantation, immunotherapy and molecularly targeted therapy are the common methods for the treatment of HCC [2, 3]. However, these methods usually cause side effects and toxicity toward normal tissues, which could not improve the overall survival of HCC patients [4]. Therefore, it is imperative to discover innovative treatment strategies for effective HCC treatment.

Nanomaterials-based photothermal therapy (PTT) has emerged as a gentle cancer therapeutic technique without serious adverse effects [5–7]. In principle, photosensitizing agents absorb light energy and convert them into thermal energy to destroy tumors [8]. Compared with other tumor treatment methods, PTT has its intrinsic advantages, such as noninvasive, high spatiotemporal selectivity and low toxicity to normal cells and tissues [9]. Nanoparticles (NPs) with strong near-infrared (NIR) region absorption ability and favorable photothermal conversion efficiencies have been designed for PTT applications [10]. Many types of Au-based plasmonic NPs, such as nanorods, nanospheres, nanostars, nanoshells and nanocages have been widely used for PTT of cancer [11–13]. Gold nanostars (AuNSs) have attracted great attention in various biomedical applications because of their optical extinction coefficients, well-established biocompatibility and localized surface plasmon resonance band that could be tuned into the NIR region [14]. AuNSs have the characteristics of irregular shape and high surface-to-volume ratio, which show high photothermal transduction efficiency due to the electric field penetrates into the nanostructures more easily [15]. Therefore, AuNSs have been widely used as optical diagnostic imaging and photothermal therapeutic agents [16].

Dopamine is a type of small-molecule compound mimic of mussel-inspired adhesive protein, and it can undergo self-polymerization and spontaneous deposition onto many materials at alkaline pH conditions, forming a designable and robust adherent polydopamine (PDA) layer [17–19]. Recently, NPs with PDA coating have received extensive attention in biological applications [20]. The PDA coating features several advantages, including good biocompatibility, and unique adhesive ability [21]. Furthermore, the outer PDA surface contains numerous amino and catechol groups, allowing for easy functionalization with cell-specific ligands, such as folic acid, antibodies, aptamers, peptides, organic small molecules and so on. These cell-specific ligands could promote the uptake of NPs by cancer cells [22]. As a result, the delivery specificity and efficiency of the NPs could be improved. The Arg-Gly-Asp (RGD) peptides can specifically bind to various surface integrins α_v_β_3_, which is highly overexpressed on the surface of several types of cancer, including HCC, ovarian cancers and so on [23]. A wide range of RGD peptide-conjugated nanomaterials had been prepared for the targeting imaging and therapy of tumors [24–26].

**Scheme 1.**
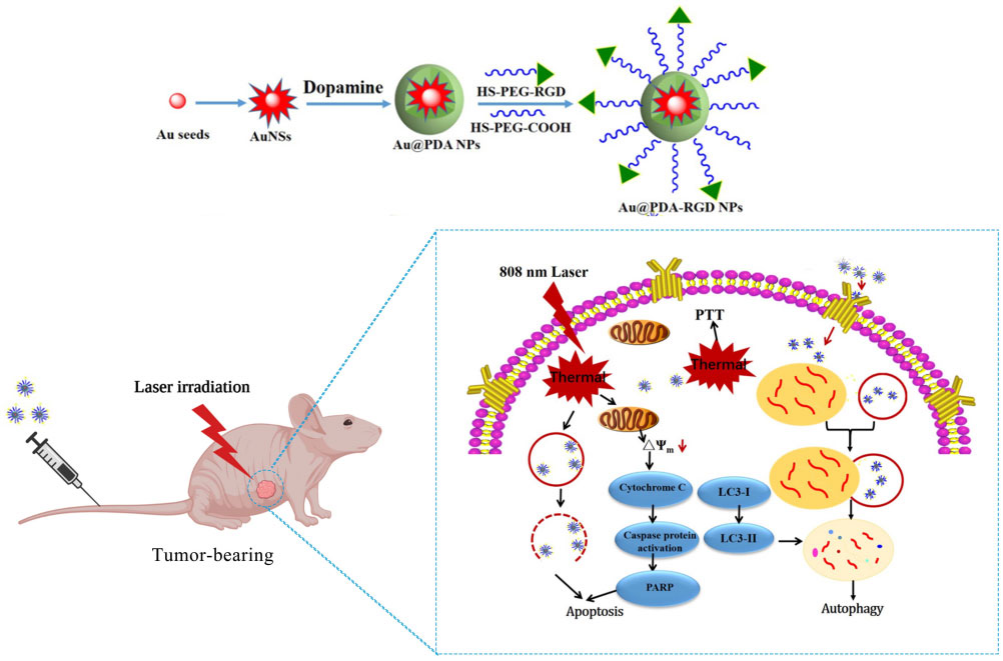
Schematic illustration of the preparation for the Au@PDA-RGD NPs and its application in targeted PTT of HCC.

In this work, RGD peptide-conjugated PDA-coated AuNSs NPs (Au@PDA-RGD NPs) were prepared for targeted PTT of HCC (Scheme 1). The as-prepared nanocomposites featured a PDA shell and AuNSs core. Carboxyl–polyethylene glycol thiol (SH-PEG-COOH, Mw 5000) and RGD-terminated polyethylene glycol thiol (SH-PEG-RGD, Mw 5000) were covalently conjugated to the surface of Au@PDA NPs [27], which could improve the biocompatibility and targeting capabilities of the nanocomposites. In this way, Au@PDA-RGD NPs were obtained. The as-prepared nanocomposites showed high photothermal conversion efficiency and good biocompatibility. The bio-transmission electron microscopy (Bio-TEM) and confocal laser scanning microscope (CLSM) images results showed the Au@PDA-RGD NPs could be effectively internalized by HepG2 cells because the RGD peptide could specifically target the α_v_β_3_ receptors on HepG2 cells [28]. The *vitro* experiment showed the Au@PDA-RGD NPs could induce the apoptosis of HepG2 cells under the irradiation of a single-wavelength laser (808 nm). The PTT mechanism was also evaluated by investigating the therapy influence on mitochondrial membrane potential, lysosomes integrity and autophagy. Au@PDA-RGD NPs could also be used for tumor-bearing nude mice PTT treatment without any obvious side effects. This nanoplatform provides an exciting strategy to treat HCC by Au@PDA-RGD NPs-mediated PTT.

## Materials and methods

### Reagents and materials

Silver nitrate (AgNO_3_), chloroauric acid tetrahydrate (HAuCl_4_·4H_2_O), ascorbic acid (AA), tris(hydroxymethyl)aminomethane (Tris), Rhodamine 6G (Rh6G) and trisodium citrate dehydrate (Na_3_C_6_H_5_O_7_·2H_2_O) were all purchased from Sinopharm Chemical Reagent Co., Ltd. Carboxyl–polyethylene glycol thiol (SH-PEG-COOH, Mw 5000) and RGD-terminated polyethylene glycol thiol (SH-PEG-RGD, Mw 5000) were obtained by Shanghai ToYongBio Tech. Inc. Dopamine hydrochloride, acridine orange (AO), tween 20, trypan blue, calcein-AM and propidium iodide (PI) were purchased from Sigma-Aldrich. Mitochondrial membrane potential assay kit with JC-1, HRP-labeled goat anti-rabbit IgG and 4',6-diamidino-2-phenylindole (DAPI) were available from Beyotime Biotechnology (China). Cell Counting Kit-8 (CCK-8) was purchased from Dojindo Molecular Technologies (Japan). Fetal bovine serum (FBS) and 0.25% trypsin-EDTA (1×) were obtained from Gibco Life Technologies. Phosphate buffered solution (PBS) and Dulbecco’s Modified Eagle Medium (DMEM) were purchased from HyClone Lab., Inc. Annexin V-PE Apoptosis Detection Kit I was purchased from BD Biosciences. All commercial chemicals were used as received without further purification. Primary antibodies against poly ADP-ribose polymerase (PARP), caspase3, caspase8, caspase9, p62, light chain 3 (LC3)B and β-actin were acquired from Cell Signaling Technology, Inc. Deionized water (18.2 MΩ·cm) was used throughout the experiments.

### Preparation of Au@PDA-RGD NPs

PDA-coated AuNSs (Au@PDA NPs) were prepared following our previous work with some adjustments [29]. PEGylated AuNSs were first prepared according to previous reports [30]. Briefly, 400 μl of citrate-stabilized seed solution was added to 40 ml of HAuCl_4_ solution (0.25 mM) under moderate stirring. Then, 400 μl of AgNO_3_ (2 mM) and 200 μl of AA (100 mM) were quickly mixed with the above solution under stirring for 1 min to obtain AuNSs. PEGylated AuNSs were prepared by adding SH-PEG-COOH (1 mg/ml) into the freshly prepared AuNSs overnight followed by centrifugation at 8000 rpm for 15 min at 4°C.

To preparation of AuNSs@PDA NPs, PEGylated AuNSs were incubated with dopamine solution in tris buffer (10 mM, pH 8.5) containing 0.02% tween 20 for 3 h with rotation at room temperature. The concentration of dopamine was 0.6 mg/ml. The Au@PDA NPs were collected by centrifugation at 8000 rpm for 15 min at 4°C. To increase the biocompatibility and targeting capabilities, PEG and RGD peptides were grafted to the surface of Au@PDA NPs. Briefly, the mixture of SH-PEG-COOH and SH-PEG-RGD with molar ratio of 1:3 was added to the solution of Au@PDA NPs at room temperature. After stirring for 24 h, Au@PDA-RGD NPs were obtained by centrifugation and washed three times with water.

### Characterization

The ultraviolet-visible-near infrared (UV-vis-NIR) absorption spectra of Au@PDA NPs and Au@PDA-RGD NPs were measured using a HITACHI UH4150 UV-vis-NIR spectrophotometer. The shape and morphology of Au@PDA NPs and Au@PDA-RGD NPs were obtained by TEM images using a Tecnai G220 transmission electron microscope. The powder X-ray diffraction (XRD) patterns of the products were carried out on a Bruker D8 advanced powder X-ray diffractometer with graphite monochromatized Cu Kα radiation. The particle size (diameter, nm) and zeta potential (potential, mV) were measured using a Zetasizer-Nano ZS from Malvern Instruments. Thermogravimetric analysis (TGA) was performed on a thermo gravimetric analyzer (Q500) under an air atmosphere at a heating rate of 10°C min^−1^ from 25 to 900°C. The photothermal heating effect of Au@PDA-RGD NPs was investigated using the CW diode laser (808 nm) and an infrared thermal imaging camera (Testo 865, IRS, Germany). The intracellular uptake of Au@PDA-RGD NPs was evaluated using Bio-TEM (JEM-1200EX). The quantitatively determine the uptake of Au in HepG2 cells was measured by inductively coupled plasma mass spectrometry (ICP-MS).

### Cell culture

Human HCC cells (HepG2) were purchased from ATCC (Manassas, VA) and cultured in DMEM. The cell culture medium was supplemented with 10% FBS, 100 U/ml penicillin and 0.1 mg/ml streptomycin. The cells were maintained in humidified conditions (37°C, 5% CO_2_).

### Cytotoxicity study

HepG2 cells were seeded in a 96-well plate and allowed to attach at 37°C under 5% CO_2_ for 24 h. Then the predetermined concentrations of Au@PDA-RGD NPs (0, 40, 80, 120, 160, 200 and 240 μg/ml) in culture media were added. The cells were incubated with Au@PDA-RGD NPs for 24 h. The cells treated with culture media alone were used as control. After that, the media were removed, and each well was washed once with cold PBS. Afterward, CCK-8 working solution was added into each well and the culture plate was incubated for an additional 2 h. A microplate reader was used to measure the absorbance at 450 nm. Each experiment was performed in triplicate.

### Cellular uptake measurement

To observe the cellular uptake of Au@PDA-RGD NPs, HepG2 cells were incubated with Au@PDA-RGD NPs for 24 h. Then, the cells were rinsed with PBS for three times and further trypsinized for 3 min. Afterward, cells were detached and collected by centrifugation. The collected cells were then fixed with 2.5% glutaraldehyde. After that, the cells were washed with PBS and dehydrated using ethanol with series concentrations. Finally, the samples were embedded and sliced for TEM (JEM-1200EX) observation to visualize the distribution of Au@PDA-RGD NPs inside HepG2 cells. In order to evaluate the internalization of Au@PDA-RGD NPs by fluorescence, the Au@PDA-RGD NPs were then labeled with Rh6G. Rh6G-labeled Au@PDA-RGD NPs were synthesized by the following method. First, AuNSs were labeled with Rh6G by incubating AuNSs (40 ml) with Rh6G (1 mM) and SH-PEG-COOH (1 mg/ml) solution overnight to obtain Rh6G-AuNSs. Then a layer of PDA was coated on the surface of Rh6G-AuNSs. Finally, SH-PEG-COOH and SH-PEG-RGD were modified on the surface of PDA-coated Rh6G-AuNSs. Rh6G-labeled Au@PDA-RGD NPs were collected by centrifugation. Rh6G-labeled Au@PDA-RGD NPs were incubated with HepG2 cells for 2 h or 4 h. Thereafter, cells were fixed using 4% paraformaldehyde, and co-incubation with DAPI for nucleus staining, followed by washing with chilled PBS (4°C). Following this, coverslips were taken out from the wells and mounted onto glass slides. The intracellular Au@PDA-RGD NPs were visualized by CLSM (Leica sp5) using 630 nm emission filter for R6G and 488 nm emission filter for DAPI.

To quantitatively the uptake of Au@PDA-RGD NPs into HepG2 cells, the amount of Au was determined by ICP-MS. Briefly, 3 × 10^5^ cells per well were seeded in six-well plates at 37°C with 5% CO_2_ for 24 h. Cells were further incubated with Au@PDA-RGD NPs for different times. Plates were then washed twice with PBS, trypsinized, collected and digested with aqua regia. Each sample was made up to 25 ml with ultra-pure water for ICP-MS measurements.

### *In vitro* photothermal toxicity of Au@PDA-RGD NPs

The photothermal cytotoxicity of the Au@PDA-RGD NPs was first assessed using the standard CCK-8 method. Briefly, HepG2 cells were seeded in 96-well plates and incubated with Au@PDA-RGD NPs (240 μg/ml) for 24 h, followed by 808 nm laser irradiation (2.0 W/cm^2^ for 5 min). HepG2 cells in the control group received the same irradiation. After a further incubation for 24 h, the viabilities of HepG2 cells were evaluated using the CCK-8 method.

HepG2 cells were seeded in 24-well plates and cultured for 24 h. The photothermal toxicity experiment was divided into four groups (control, control + laser, Au@PDA-RGD NPs and Au@PDA-RGD NPs + laser). The cells had no treatment in the control group. The cells were irradiated with an 808 nm laser at 2.0 W/cm^2^ for 5 min for the control + laser group. For the Au@PDA-RGD NPs and Au@PDA-RGD NPs + laser group, HepG2 cells were incubated with Au@PDA-RGD NPs for 24 h, and then the cells were illuminated without or with 808 nm laser (2.0 W/cm^2^) for 5 min, respectively. After laser irradiation treatment, the old medium was replaced with fresh DMEM, and cells were incubated overnight. Cells were then washed gently with PBS and imaged immediately using an inverted light microscope (Olympus BX41) in a bright field or after additional staining. Trypan blue staining was used to determine the level of cell membrane damage. Briefly, 0.4% trypan blue solution was added to the treated HepG2 cells and incubated for 5 min. Then trypan blue was washed by PBS and cells were examined on an inverted light microscope (Olympus BX41) in a bright field. The viable cells remained clear, while dead cells were stained with blue color [31]. Calcein-AM (live cells, green emission) and PI (dead cells, red emission) double staining were performed to visualize the live and dead cells. After treatment in different ways, HepG2 cells were stained with calcein-AM/PI for 15 min at 37°C in the dark. Then, the culture medium was discarded and cells were washed with PBS (pH 7.4). An inverted fluorescence microscope (Nikon Eclipse Ti-E) was used to record the images.

To detect the apoptosis-inducing capabilities of Au@PDA-RGD NPs before and after the 808 nm laser irradiation, the Annexin V-PE Apoptosis Detection Kit I was used to evaluate the cell apoptosis and necrosis ratios. HepG2 cells were seeded in 24-well plates at a density of 2 × 10^5^ cells/well. After respective treatments, HepG2 cells in each group were trypsinized and re-suspended in 1× binding buffer. Afterward, cells were stained with Annexin V-PE and 7-AAD. Finally, the cell apoptosis and necrosis of the samples were quantitatively analyzed by flow cytometer (BD FACS Aria III).

### Mitochondrial membrane potential and lysosomal membrane integrity analysis

Changes in mitochondrial membrane potential (△Ψm) were measured using JC-1 as a fluorescent probe [32]. Briefly, after respective treatments, HepG2 cells were stained with JC-1 (10 μg/ml) at 37°C for 20 min in the dark. After removing the JC-1 solution and washing with PBS, changes in △Ψm were visualized by an inverted fluorescence microscope (Nikon Eclipse Ti-E). For a quantitative JC-1 assay, the treated HepG2 cells were gently collected and stained with JC-1. The cells were then re-suspended in PBS and cell-associated fluorescence was assayed using flow cytometry.

Lysosomal membrane permeability (LMP) was assessed using the AO retention assay [33]. Briefly, after respective treatments, HepG2 cells were gently collected and incubated with AO (5 μg/ml) at 37°C for 30 min, and then the flow cytometry was used to detect the red fluorescence intensity.

### Autophagy induced by the PTT

HepG2 cells were seeded onto 24-well plates and incubated for 24 h. The medium was removed and replaced by DMEM or Au@PDA-RGD NPs for another 24 h incubation. After respective treatments, the media were removed, and each well was washed once with cold PBS. Afterward, the cells were stained with monodansylcadaverine (MDC, an autophagic vacuole-specific fluorescence probe) solution and subjected to BD FACS Aria III flow cytometer. Bio-TEM was also used to observe the autophagosomes in HepG2 cells.

### Western blotting assay

HepG2 cells with various treatments were harvested and homogenized in ice-cold radioimmunoprecipitation assay buffer. The protein concentration in all samples was determined using the classic bicinchoninic acid (BCA) assay. Equal amounts of the extracted protein samples were fractionated by a 10% sodium dodecyl sulfate-polyacrylamide gel electrophoresis and then electroblotted onto a polyvinylidene fluoride (PVDF) membrane. The PVDF membrane was blocked with 5% non-fat milk in TBST (20 mM Tris-HCl, 150 mM NaCl, 0.05% Tween 20, pH 8.0) buffer. And then the membrane was probed with primary antibodies against PARP, caspase3, caspase8, caspase9, p62 and LC3B overnight at 4°C. β-actin was used as an internal standard for loading. Blotted proteins were detected using ECL reagents (Beyotime Biotechnology) after incubated with HRP-labeled secondary antibodies.

### *In vivo* PTT antitumor efficacy and safety evaluation

Female 5–6-week-old BALB/c nude mice were obtained from Jinan Pengyue Experimental Animal Breeding Co., Ltd. We confirm that all experimental protocols were approved by the Institutional Animal Care and Use Committee of Liaocheng People’s Hospital. The PTT therapeutic efficacy of Au@PDA-RGD NPs was assessed in subcutaneous HepG2 tumor-bearing nude mice. HepG2 cells (5 × 10^6^ cells/mouse) were implanted subcutaneously into the right axillary fossa in the mice. When the tumor volume reached approximately 120 mm^3^, mice were randomly divided into four groups (*n* = 5): (a) PBS, (b) PBS + laser, (c) Au@PDA-RGD NPs and (d) Au@PDA-RGD NPs + laser, respectively. The mice were intravenous injected with 100 μl of PBS or Au@PDA-RGD NPs at a dose of 10 mg/kg. After 6 h of injection, the mice in Groups b and d were irradiated with NIR laser (808 nm, 0.8 W/cm^2^) for 5 min. The temperature variation and thermal images of these mice were recorded using the infrared thermal imaging camera (Testo 865, IRS, Germany). The tumor volume and weight of mice were measured every other day until the end of experiments on Day 14. The tumor volume (V) was calculated using the formula: V (mm^3^) = (Tumor length × Tumor width^2^)/2. V/V_0_ (V_0_ is the tumor volume when the treatment was initiated) was denoted as the relative tumor volume. In order to determine the side effects of Au@PDA-RGD NPs-mediated PTT *in vivo*, the blood samples of mice were collected for blood routine examination and serum biochemistry study after the experiments, and the major organs were made into paraffin sections for further hematoxylin and eosin (H&E) staining following manufacturers’ protocol.

## Results and discussion

### Synthesis and characterization of Au@PDA-RGD NPs

**Figure 1.**
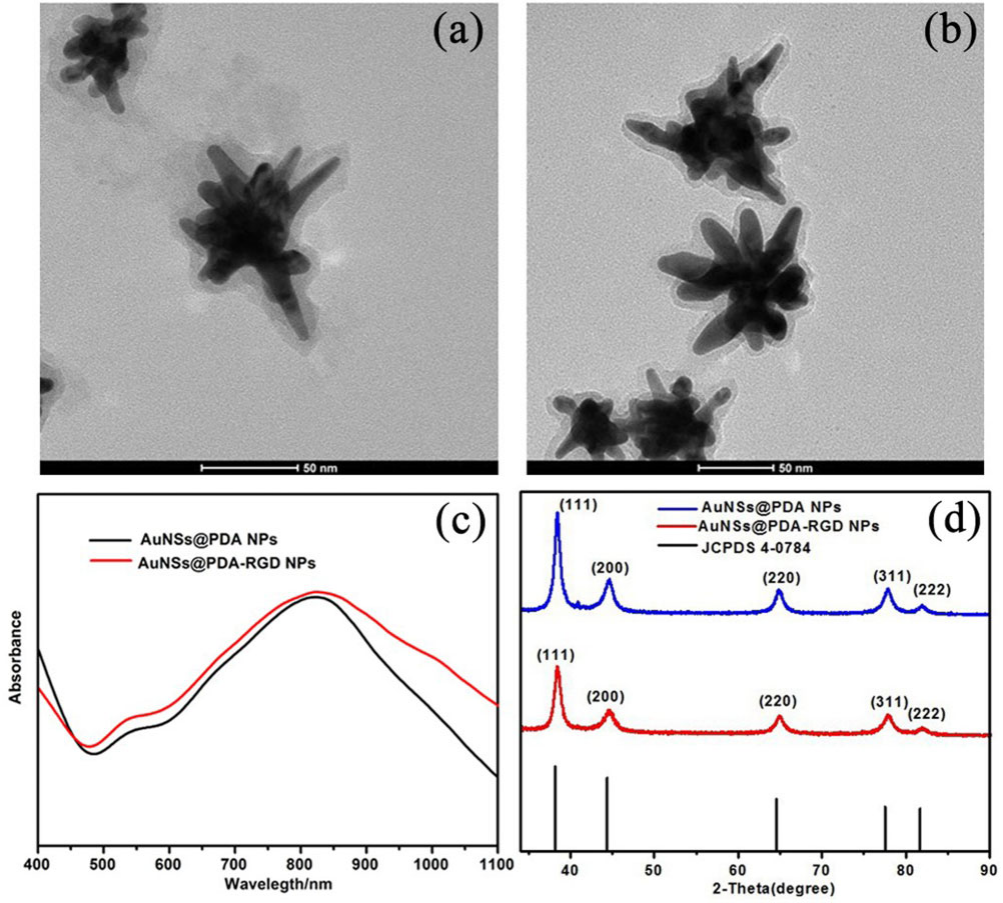
Characterization of Au@PDA-RGD NPs. Transmission electron microscopy (TEM) images of Au@PDA NPs (**a**) and Au@PDA-RGD NPs (**b**). (**c**) UV-vis-NIR spectra of Au@PDA NPs and Au@PDA-RGD NPs. (**d**) XRD patterns of Au@PDA NPs and Au@PDA-RGD NPs.

In our study, AuNSs were prepared first by the surfactant-free synthesis method according to previous reports [29]. To avoid aggregation, the AuNSs were modified with a layer of PEG. Second, in an alkaline environment, the dopamine catechol is first oxidized to quinone, which further reacts with other catechols and/or quinones to form a layer of PDA shell on the surface of PEGylated AuNSs (Au@PDA NPs) (Fig. 1a) [15]. To improve the stability and provide HCC-targeting ability, Au@PDA NPs were further modified with SH-PEG-COOH and SH-PEG-RGD to form Au@PDA-RGD NPs via Michael addition or Schiff base formation (Fig. 1b). The diameter distribution and zeta potentials of Au@PDA NPs and Au@PDA-RGD NPs were detected using by the Zetasizer-Nano ZS. The measurement results showed that the surface modification did not significantly alter the diameter and surface charge (Supplementary Figs S1 and S2). We further investigate the colloidal stability of AuNSs@PDA-RGD NPs by exposing them to water, PBS buffer or cell culture medium (DMEM) (Supplementary Fig. S3). It was observed that the Au@PDA-RGD NPs showed good chemical stability for at least 24 h without the formation of sedimentation. The optical properties of the NPs were also studied by UV-vis-NIR spectrophotometer. As shown in Fig. 1c, the maximum absorption wavelengths for Au@PDA-RGD NPs was about 822 nm. The absorption spectrum of Au@PDA-RGD NPs located within the NIR region (700–1100 nm), confirming they could be used as potential photothermal conversion agents for PTT. The power XRD patterns of Au@PDA NPs and Au@PDA-RGD NPs showed the characteristics peaks of the (111), (200), (220), (311) and (222) planes (Fig. 1d), which matched well with the face-centered cubic Au structure (JCPDS No. 4-0784). Supplementary Fig. S4 showed the FTIR spectra of Au@PDA NPs and Au@PDA-RGD NPs. The characteristic peaks of amide III (1382 cm^−1^) appeared in Au@PDA-RGD NPs [34, 35], demonstrated the successful conjugation of RGD on the Au@PDA NPs. TGA was also conducted to directly measure the weight loss of PEGylated AuNSs, Au@PDA NPs and Au@PDA-RGD NPs. As shown in Supplementary Fig. S5, the weight loss of the PEGylated AuNSs was about 11.1 wt%. Meanwhile, the weight loss was nearly 30.6 wt% for Au@PDA NPs and 46.3 wt% for Au@PDA-RGD NPs, respectively, indicating that ∼19.5 wt% of PDA and ∼15.7 wt% of SH-PEG-COOH and SH-PEG-RGD were introduced onto the surface of the PEGylated AuNSs. The TGA results further illustrated that the PDA and RGD had been successfully loaded on the surface of the PEGylated AuNSs.

### Evaluation of photothermal effects of Au@PDA-RGD NPs

**Figure 2.**
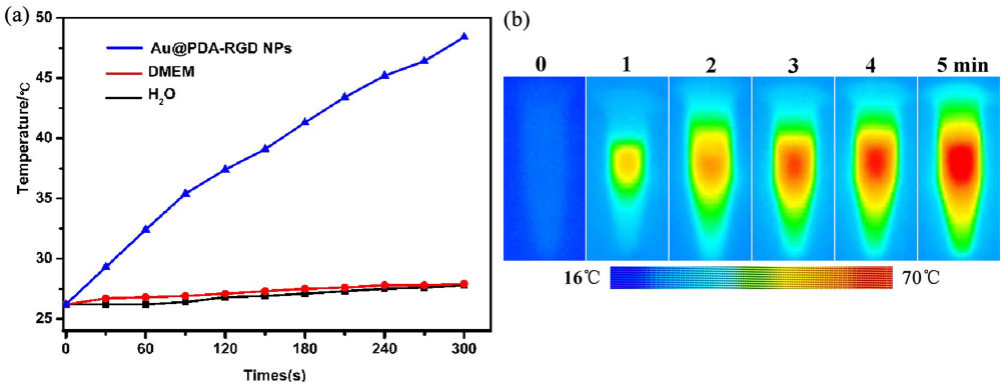
(**a**) Temperature elevation profiles of Au@PDA-RGD NPs solution under the laser irradiation, DMEM and H_2_O were used as the negative control. (**b**) Infrared thermal images of Au@PDA-RGD NPs solution under continuous irradiation.

To confirm the photothermal effects of Au@PDA-RGD NPs, the photothermal conversion capabilities were evaluated. The changes in samples temperature were monitored under a diode laser irradiation at 808 nm. Fig. 2a shows the temperature profile of Au@PDA-RGD NPs (240 μg/ml, 2.0 W/cm^2^). The temperature of Au@PDA-RGD NPs rapidly increased from 26.2–48.4°C within 5 min, confirming the capability of Au@PDA-RGD NPs to convert NIR light energy to heat. In contrast, the temperature of DMEM and H_2_O was only increased about 2°C under the same irradiation conditions. To intuitively illustrate the photothermal conversion characteristic of Au@PDA-RGD NPs, the real-time infrared thermal images were recorded (Fig. 2b). The UV-vis-NIR absorption spectrum and TEM images of Au@PDA-RGD NPs were also measured after laser irradiation (Supplementary Fig. S6), both of which exhibited no obvious change, indicating the photostability of Au@PDA-RGD NPs.

### Cellular uptake

It has been reported that RGD-modified NPs could be internalized by cells via receptor-mediated endocytosis [23]. ICP-MS was employed to verify the specific targeting capability of Au@PDA-RGD NPs to HepG2 cells. HepG2 cells were treated with Au@PDA-RGD or Au@PDA-COOH for different times, and the amount of internalized elemental Au was investigated by ICP-MS. As shown in Supplementary Fig. S7, for the Au@PDA-RGD NPs or Au@PDA-COOH NPs, the uptake of elemental Au depended on the incubation time. Moreover, significant amounts of internalized elemental Au were detected for HepG2 cells treated with Au@PDA-RGD NPs. These phenomena indicated that Au@PDA-RGD NPs had good targeting capability toward HepG2 cells.

**Figure 3.**
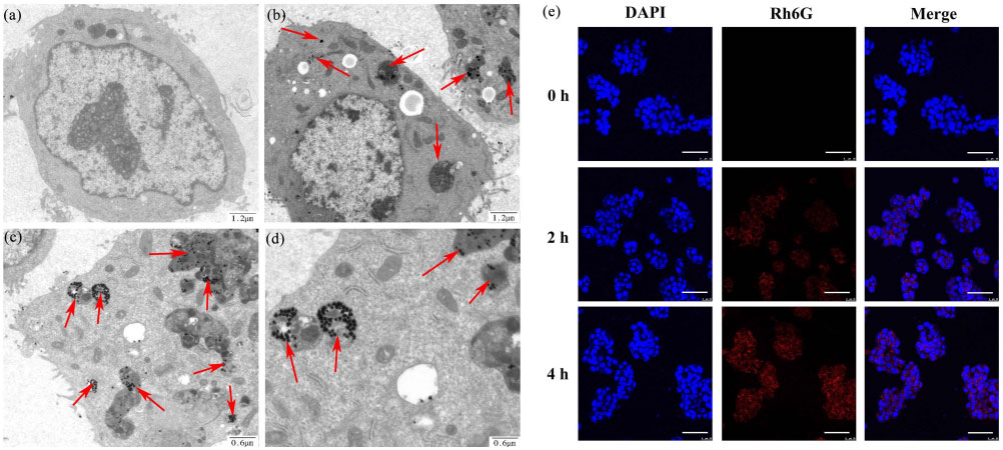
Representative TEM section images of HepG2 cells incubated with (**a**) DMEM or (**b**–**d**) Au@PDA-RGD NPs. (**e**) CLSM images of HepG2 cells incubated with Rh6G-labeled Au@PDA-RGD NPs for 0, 2 and 4 h (scale bar is 50 μm).

Bio-TEM was used to observe the ultrastructure of HepG2 cells to prove Au@PDA-RGD NPs had entered into the cellular. As shown in Fig. 3b– d, Au@PDA-RGD NPs is clearly visible in the cells and could be easily distinguished from the cellular as gold has high electron density. The shape and size of Au@PDA-RGD NPs had negligible change after being endocytosed in cells. We infer that the Au@PDA-RGD NPs can target the integrin receptor on the surface of HepG2 cells, and mainly internalized through integrin receptor-mediated endocytosis. In the control HepG2 cells (Fig. 3a), normal cell morphology is observed [28]. The CLSM images (Fig. 3e) demonstrated that HepG2 cells incubated with the Rh6G-labeled Au@PDA-RGD NPs exhibited red fluorescence owing to their efficient cellular internalization, which showed time-dependent.

### *In vitro* PTT effects of Au@PDA-RGD NPs

**Figure 4.**
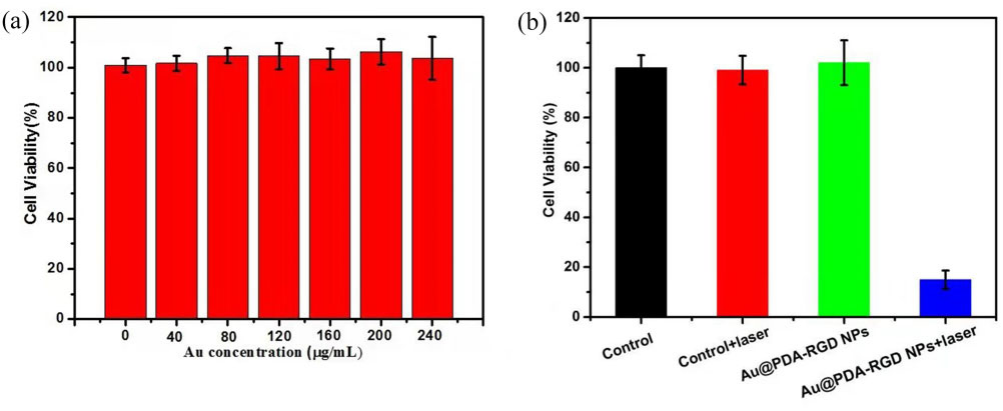
(**a**) CCK-8 assay of HepG2 cells after being co-cultured with Au@PDA-RGD NPs with the Au concentrations of 0–240 μg/ml for 24 h. (**b**) Relative viabilities of HepG2 cells after Au@PDA-RGD NPs treatment with or without laser illumination.

To assess the biocompatibility of Au@PDA-RGD NPs on cancer cells, HepG2 cells were incubated with different concentrations of Au@PDA-RGD NPs. The CCK-8 assay revealed that Au@PDA-RGD NPs did not show toxic effect to HepG2 cells at the concentration of 0–240 μg/ml (Fig. 4a). When the concentration reaches 240 μg/ml, not obvious reduction of cell viability was observed for HepG2 cells, suggesting that Au@PDA-RGD NPs have excellent biocompatibility. The photothermal cytotoxicity of Au@PDA-RGD NPs was then detected, as shown in Fig. 4b. For the control, control + laser and Au@PDA-RGD NPs group, no cytotoxicity was observed. However, the cell viability decreased to about 18% for the Au@PDA-RGD NPs + laser group because of the photothermal killing of Au@PDA-RGD NPs excited by laser irradiation.

**Figure 5.**
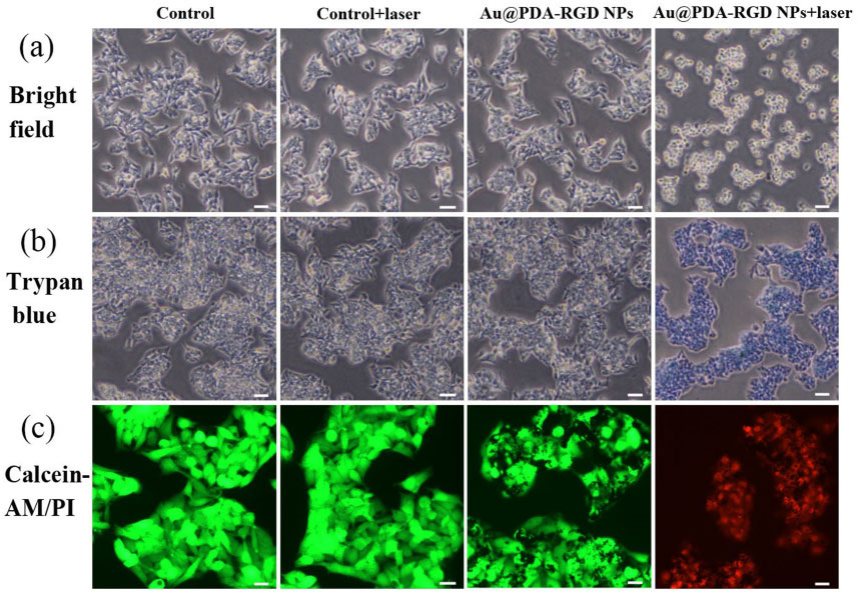
HepG2 cells treated with or without Au@PDA-RGD NPs under 808 nm NIR laser irradiation. (**a**) Morphological alterations (scale bar is 20 μm). (**b**) Trypan blue staining (scale bar is 20 μm). (**c**) HepG2 cells stained by calcein-AM (live: green) and propidium iodide (PI, dead: red) (scale bar is 10 μm).

To visually evaluate the PTT effect, the morphology changes of HepG2 cells under different treatments were observed under the optical microscope. As seen from Fig. 5a, the cells were intact and exhibited normal proliferation behavior in the control, control + laser and Au@PDA-RGD NPs group. For the Au@PDA-RGD NPs + laser group, the cells turned rounded, shrinkage, membrane integrity loss or separated from neighboring cells, demonstrating obvious apoptotic features [31, 36].

Next, trypan blue staining was performed to evaluate the PTT efficiency of Au@PDA-RGD NPs against HepG2 cells (Fig. 5b). No visible changes were observed in the control group. Moreover, no HepG2 cells were stained in the control + laser or Au@PDA-RGD NPs group, implying that the laser or Au@PDA-RGD NPs alone were biocompatible *in vitro*. In contrast, HepG2 cells were stained blue in the Au@PDA-RGD NPs + laser group, indicating the cells were dead. The trypan blue staining results were consistent with that of the CCK-8 assay.

In addition, the PTT efficiency of Au@PDA-RGD NPs was confirmed using live/dead cell staining experiments. The live cells and dead cells were stained with calcein-AM (green) and PI (red), respectively [37–39]. For the control, control + laser group and Au@PDA-RGD NPs group, vivid green fluorescence was observed in HepG2 cells, which indicated the live state of cells. In contrast, a majority of red fluorescence was observed in the Au@PDA-RGD NPs + laser group (Fig. 5c), suggesting the PTT could effectively kill the cancer cells.

**Figure 6.**
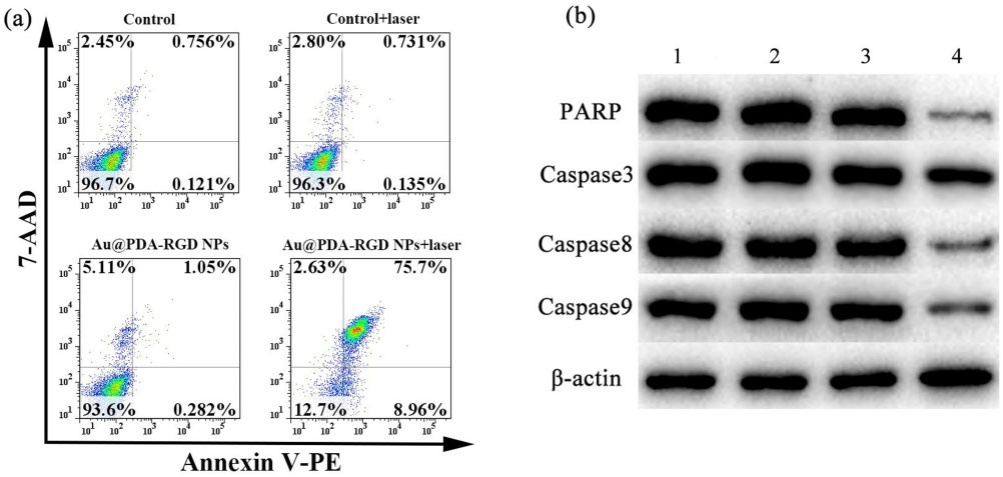
(**a**) Apoptosis results of HepG2 cells after various treatments. (**b**) Western blot analysis of PARP, caspase3, caspase8 and caspase9 in HepG2 with different treatment. Lane 1, control; lane 2, control + laser; lane 3, Au@PDA-RGD NPs; lane 4, Au@PDA-RGD NPs + laser.

To specifically quantify the viability of the cells, flow cytometry analyses were further performed. HepG2 cells were treated with Au@PDA-RGD NPs for 24 h with or without laser irradiation, and then the cytotoxicity was investigated using the Annexin V-PE and 7-AAD double staining assay by flow cytometry. Double-stained cells represented as late apoptotic [31]. It could be clearly seen from Fig. 6a that the cell viability remained at above 90% in the control, control + laser or Au@PDA-RGD NPs group. In the Au@PDA-RGD NPs + laser group, the cell viability decreased to 12.7%. The results evidently illustrated that Au@PDA-RGD NPs are efficient photothermal agents, which could obviously promote the efficiency of cell apoptosis under NIR laser irradiation.

To further identify the mechanisms underlying PTT-induced apoptosis in HepG2 cells, the expression levels of caspase3, caspase8, caspase9 and PARP were detected by western blot. As shown in Fig. 6b, no significant difference in the induction effects of apoptotic markers in HepG2 cells was observed in the control, control + laser and Au@PDA-RGD NPs group. On the contrary, Au@PDA-RGD NPs-mediated PTT decreased caspase3, caspase8 and caspase9, and PARP levels in HepG2 cells, which illustrated that Au@PDA-RGD NPs plus 808 nm laser irradiation could induce caspase-dependent apoptosis in HepG2 cells [40, 41].

### Mitochondrial membrane potential and lysosomes integrity analysis

**Figure 7.**
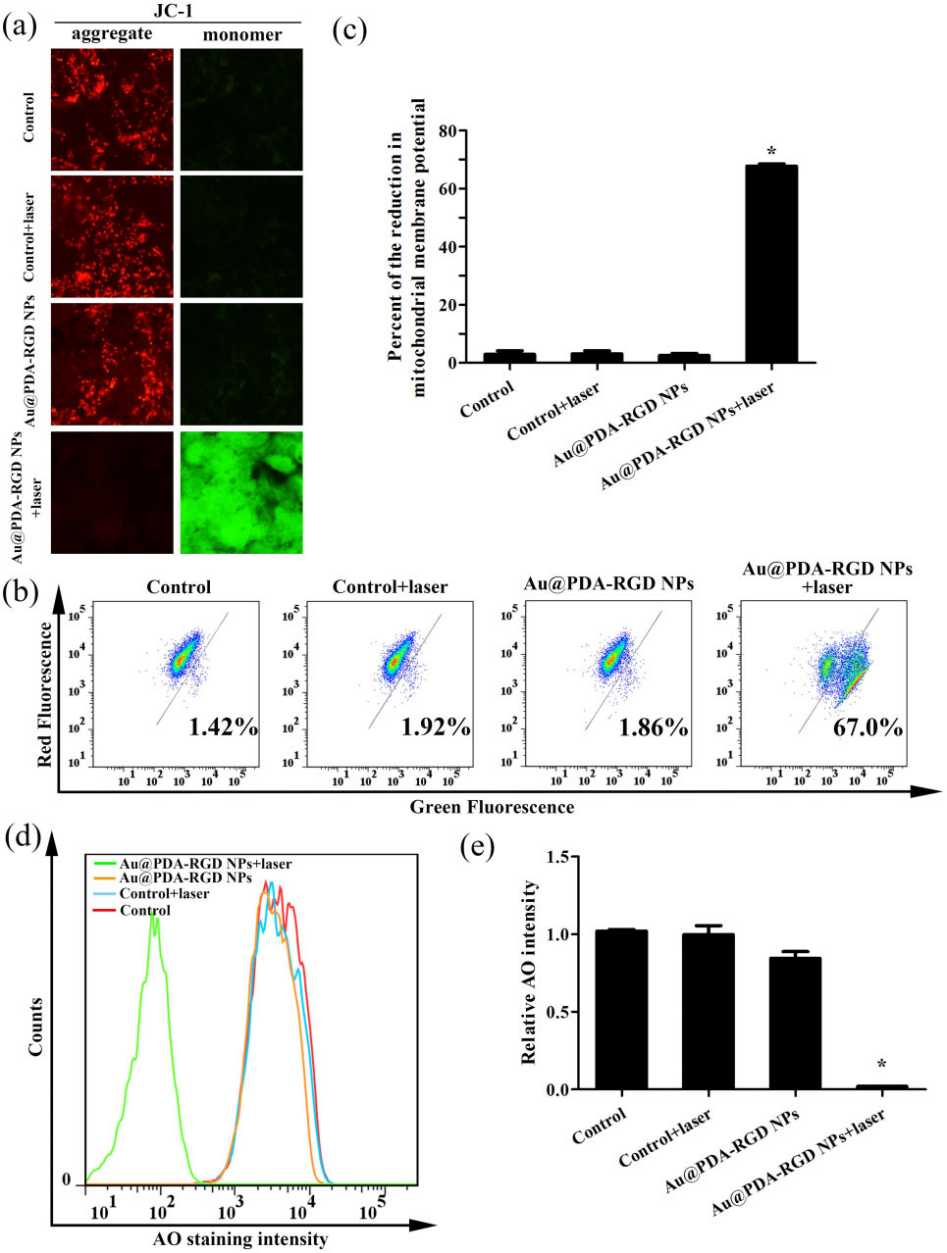
PTT-induced loss of mitochondrial membrane potential and increase of lysosomal membrane permeability in HepG2 cells. (**a**) Fluorescence imaging of the JC-1-labeled HepG2 cells (scale bar is 10 μm). (**b**) Flow cytometry assay of mitochondrial membrane potential with JC-1 as fluorescent probe. (**c**) Quantitative analysis of fluorescent intensity from (b). Data were presented as mean ± SD (*n* = 3), Student’s *t*-test, *P <* *0.05. (**d**) HepG2 cells stained with AO for flow cytometry analysis. (**e**) Quantitative analysis of fluorescent intensity from (d). Data were presented as mean ± SD (*n* = 3), Student’s *t*-test, *P <* *0.05.

The reduction of mitochondrial membrane potential (△Ψm) was considered to be one key indicator of cell apoptosis in cancerous and noncancerous cells [42, 43]. Herein, △Ψm of HepG2 cells after PTT was determined using JC-1 as the probe. JC-1, a fluorescent dichromatic probe, exhibits red fluorescence, as it enters and aggregates inside the healthy mitochondria [44]. Once △Ψm decreased, the dye will exhibit in monomer, no longer accumulate within the mitochondria and emits green fluorescence signal. As shown in Fig. 7a, for the control, control + laser and Au@PDA-RGD NPs group, the △Ψm was normal owing to the cells showed high red fluorescence signal and low green fluorescence signal, which indicated that the mitochondria were not damaged in these three groups. In contrast, Au@PDA-RGD NPs-mediated PTT induced the dysfunction of mitochondria, as indicated by the increased green fluorescence signal and the decreased red fluorescence signal. The △Ψm loss was also determined by the green fluorescence intensity of JC-1 using flow cytometry. Fig. 7b and c clearly showed that the green fluorescence of the JC-1 monomers increased to 67.0% for the Au@PDA-RGD NPs + laser group, illustrating the PTT could active the mitochondria-mediated apoptosis.

It has been reported that some forms of apoptosis could trigger LMP. The increase of LMP could lead to a release of cathepsins and destabilization of lysosomes [45]. We further analyzed the influence of PTT on LMP using AO staining. AO is a lysosomotropic fluorophore that exhibits red fluorescence when it accumulates within intact lysosomes, whereas it shows a diffuse green fluorescence upon redistribution from the lysosomes to the cytoplasm after the increase of LMP [46]. The LMP was also evaluated by the red fluorescence intensity of AO using flow cytometry. As shown in Fig. 7d and e, the red fluorescence of AO decreased for the Au@PDA-RGD NPs + laser group, which illustrates the PTT could trigger LMP.

### Effects of PTT on autophagy

**Figure 8.**
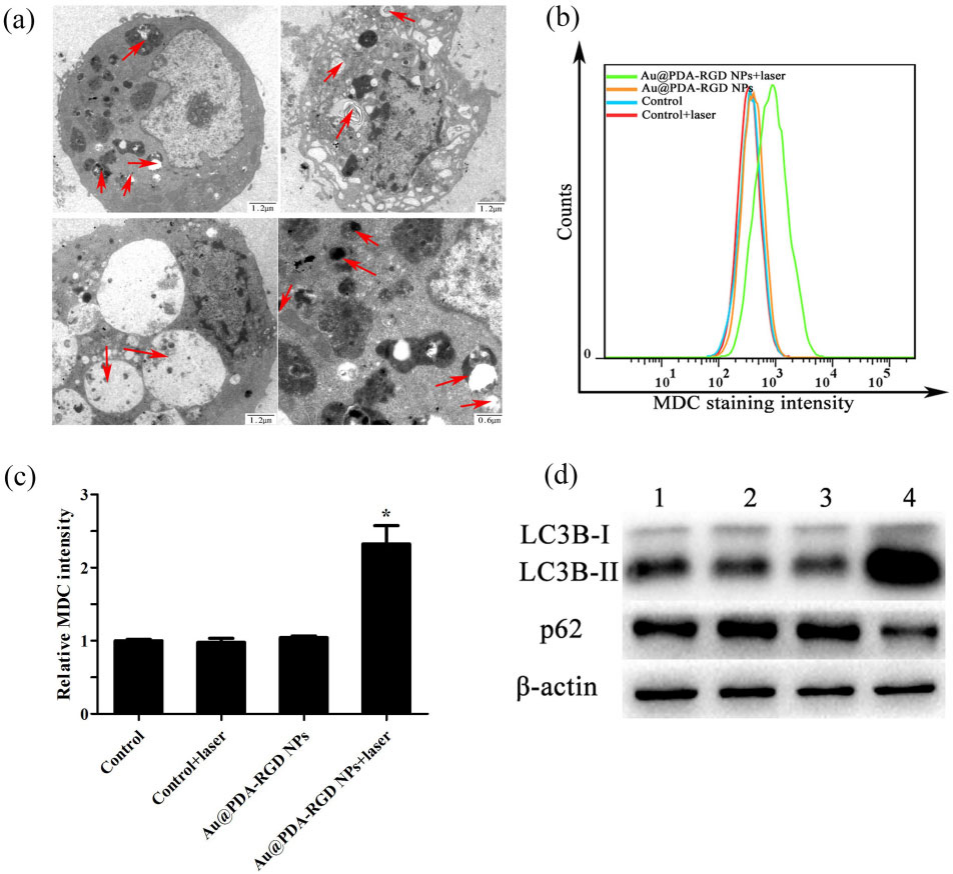
(**a**) Bio-TEM images illustrating the morphology of autophagosomes and autophagic vacuoles in HepG2 cells treated with Au@PDA-RGD NPs plus 808 nm NIR laser irradiation. Red arrows indicate some of autophagosomes and autophagic vacuoles. (**b**) Flow cytometry analysis of MDC fluorescent intensity in HepG2 cells. (**c**) Quantitative analysis of fluorescent intensity from (b). Data were presented as mean ± SD (*n* = 3), Student’s *t*-test, *P <* *0.05. (**d**) Western blot analysis of LC3B and p62 in HepG2 cells with different treatment. Lane 1, control; lane 2, control + laser; lane 3, Au@PDA-RGD NPs; lane 4, Au@PDA NPs-RGD + laser.

Autophagy, as one of the major modes of cell death besides apoptosis and necrosis [47], is considered as an important cellular process that could remove the damaged proteins and sub-cellular organelles. Autophagy exists extensively in malignant tumors [48]. To study the mechanism underlying the PTT effect, the autophagy modulation study triggered by the PTT-induced cell death was performed. Bio-TEM can be used to identify autophagosomes in cells [49]. As shown in Fig. 8a, the treated HepG2 cells exhibited large number of vacuoles (autophagosomes), which clearly illustrated that PTT could induce autophagy. MDC staining was used to detect the autophagic activity of the treated HepG2 cells. MDC is an autofluorescent base and autophagic vacuole-specific fluorescent probe, and its fluorescent could be detected by the flow cytometer [50, 51]. As shown in Fig. 8b and c, exposure of HepG2 cells to the Au@PDA-RGD NPs and laser irradiation led to an increase of MDC fluorescent intensity in comparison with other groups, illustrating the increasing number of autophagic cells. Moreover, autophagy was further detected by western blot analysis. Microtubule-associated protein 1 LC3 is a marker of autophagosomes. The LC3B-I (cytosolic form) and the LC3B-II (lapidated form) are the two present forms of LC3B [52]. LC3B-II is a molecular marker of autophagosomes, and the increased ratio of LC3B-II to LC3B-I could be used to detect autophagy. Sequestosome 1 (P62/SQSTM1) is a stress-inducible protein that can be selectively interacted with autophagosomes [40]. The expression levels of LC3B and p62-induced PTT were quantified investigated by western blot. As shown in Fig. 8d, the obviously high levels of LC3B and the reduction of p62 expression in the Au@PDA-RGD NPs + laser group suggested the autophagy process occurs. The above results suggest that the PTT can induce autophagy.

### *In vivo* PTT antitumor efficacy of Au@PDA-RGD NPs

**Figure 9.**
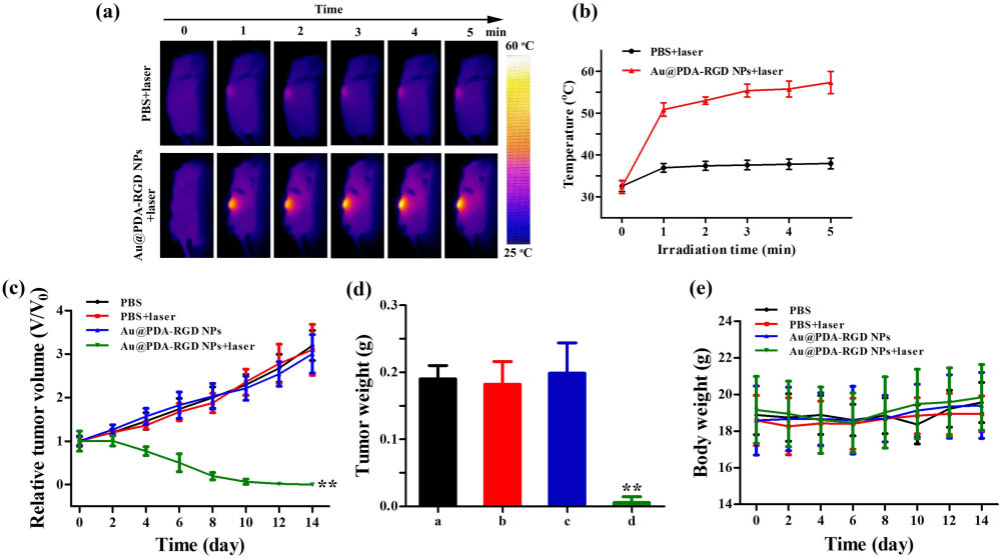
(**a**) IR thermal images of HepG-2 tumor-bearing mice exposed to the 808 nm laser irradiation (0.8 W/cm^2^, 5 min). (**b**) The temperature curves at tumor site under the 808 nm laser irradiation (0.8 W/cm^2^, 5 min). (**c**) The relative tumor volume after different treatments. (**d**) Tumor weight in different groups. (**e**) Change of body weight in different groups. Data were presented as mean ± SD (*n* = 5), Student’s t-test, **P <* *0.01.

We next performed the *in vivo* photothermal imaging and PTT capability of Au@PDA-RGD NPs on HepG2 tumor-bearing mice. As illustrated in Fig. 9a and b, the tumor temperature in Au@PDA-RGD NPs plus laser-treated mice is much higher than that in PBS-treated mice. The tumor temperature in Au@PDA-RGD NPs-treated mice rapidly exceeded 55°C after 5 min of laser irradiation, which could cause irreversible damage to tumor tissue. On the contrary, the tumor in PBS plus laser-treated mice had a maximum temperature of 38°C, which was insufficient to induce irreversible damage to tumor tissue. These results implied the Au@PDA-RGD NPs-based nano-platform exhibited the *in vivo* tumor sites specific hyperthermia.

The tumor sizes and weight in different groups were monitored to evaluate the antitumor efficacy. As shown in Fig. 9c, limited tumor inhibitory effects were observed in PBS, PBS + laser or Au@PDA-RGD NPs group. In contrast, the Au@PDA-RGD NPs plus laser remarkably inhibited tumor growth. In addition, the tumor was completely eliminated in the Au@PDA-RGD NPs + laser group (Supplementary Fig. S8), illustrating the excellent therapeutic effectiveness. Tumors collected from Au@PDA-RGD NPs + laser group showed the lowest weight among all groups (Fig. 9d).

**Figure 10.**
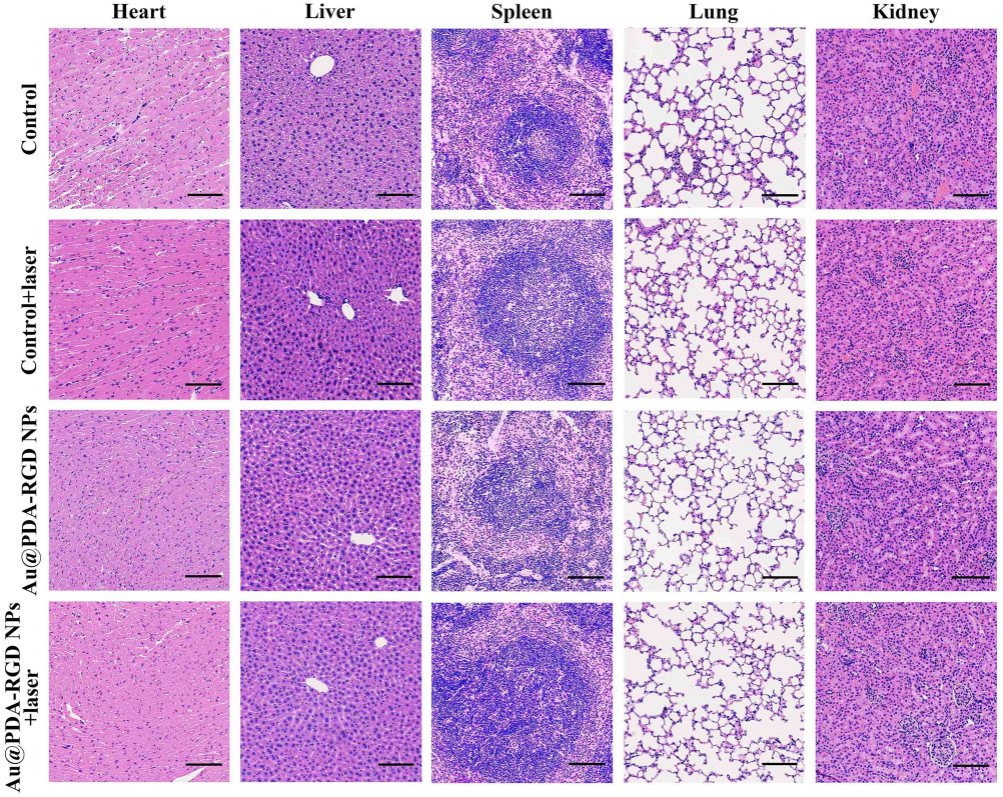
H&E staining of main organs collected from HepG2 tumor-bearing mice with various treatments (scale bar is 100 μm).

As high toxicity usually leads to weight loss, the potential biological toxicity of different treatments was also evaluated by recording the body weight of each group. As shown in Fig. 9e, the body weight showed no obvious difference in the four groups during the period of observation, implying that the treatment had good biocompatibility. Blood routine examination and blood biochemistry analysis were performed on the 14th day after treatment. All measured indicators (Supplementary Figs S9 and S10) are in the normal range. H&E staining are used to examine the potential biological toxicity of different treatments on critical organs including the heart, liver, spleen, lung and kidney. As shown in Fig. 10, neither obvious inflammation nor damage were observed in the histopathology evaluation. The above results illustrated that Au@PDA-RGD NPs-mediated PTT had favorable safety.

## Conclusions

We developed a versatile Au@PDA-RGD NPs-based nanoplatform for PTT of HCC. The targeting peptide RGD was modified onto Au@PDA NPs to achieve specific bind to various surface integrins α_v_β_3_. The obtained nanocomposites (Au@PDA-RGD NPs) showed excellent biocompatibility and receptor-mediated targeting effect toward integrins α_v_β_3_-overexpressed HepG2 cells. It was found that Au@PDA-RGD NPs plus laser irradiation could effectively induce cell damage. The Au@PDA-RGD NPs-mediated PTT induced cell death via the mitochondrial–lysosomal and autophagy pathways. *In vivo* experiments showed that Au@PDA-RGD NPs could act as PTT agents to obtain superior tumor ablation with excellent bio-security. Thus, the designed Au@PDA-RGD NPs hold a great promise to be used as effective agents for PTT of different α_v_β_3_ integrin receptor-overexpressing tumors.

## Supplementary data

Supplementary data are available at *REGBIO* online.

## Funding

The authors are grateful for financial support from National Natural Science Foundation of China (NSFC81602736), China Postdoctoral Science Foundation (2018M632684), Shandong Provincial Natural Science Foundation (ZR2019PH084) and Liaocheng People's Hospital Youth Research Fund (LYQN201935).

*Conflict of interest statement*. The authors declare no conflict of interest.

## Supplementary Material

rbab046_Supplementary_Data
