# Supplementary material for: Integrin αvβ3-targeted polydopamine-coated gold nanostars for photothermal ablation therapy of hepatocellular carcinoma
Source: Regen Biomater. 2021 Aug 10;8(5):rbab046. doi: 10.1093/rb/rbab046 (PMC8387661; doi:10.1093/rb/rbab046)
Supplement: rbab046_Supplementary_Data [file rbab046_supplementary_data.doc]

Supporting information

Integrin α_v_β_3_-targeted polydopamine-coated gold nanostars for photothermal ablation therapy of hepatocellular carcinoma

Yang Li^1^, Ping Hu^1^, Xiali Wang^3^, Xu Hou^4^, Fengzhen Liu^5^, Xiaohong Jiang^1,2^*

1 Zhong Yuan Academy of Biological Medicine, Liaocheng People's Hospital, Liaocheng 252000

2 School of Basic Medical Sciences, Shandong University, Jinan 250012

3 Clinical Laboratory, Liaocheng People's Hospital, Liaocheng 252000

4 Department of Hepatobiliary Surgery, Liaocheng People's Hospital, Liaocheng 252000

5 Liaocheng People’s Hospital, Medical College of Liaocheng University, Liaocheng 252000

*Corresponding Author: Xiaohong Jiang ([xhjiang_sd@163.com](mailto:xhjiang_sd@163.com))

Phone: +86-635-827-0102


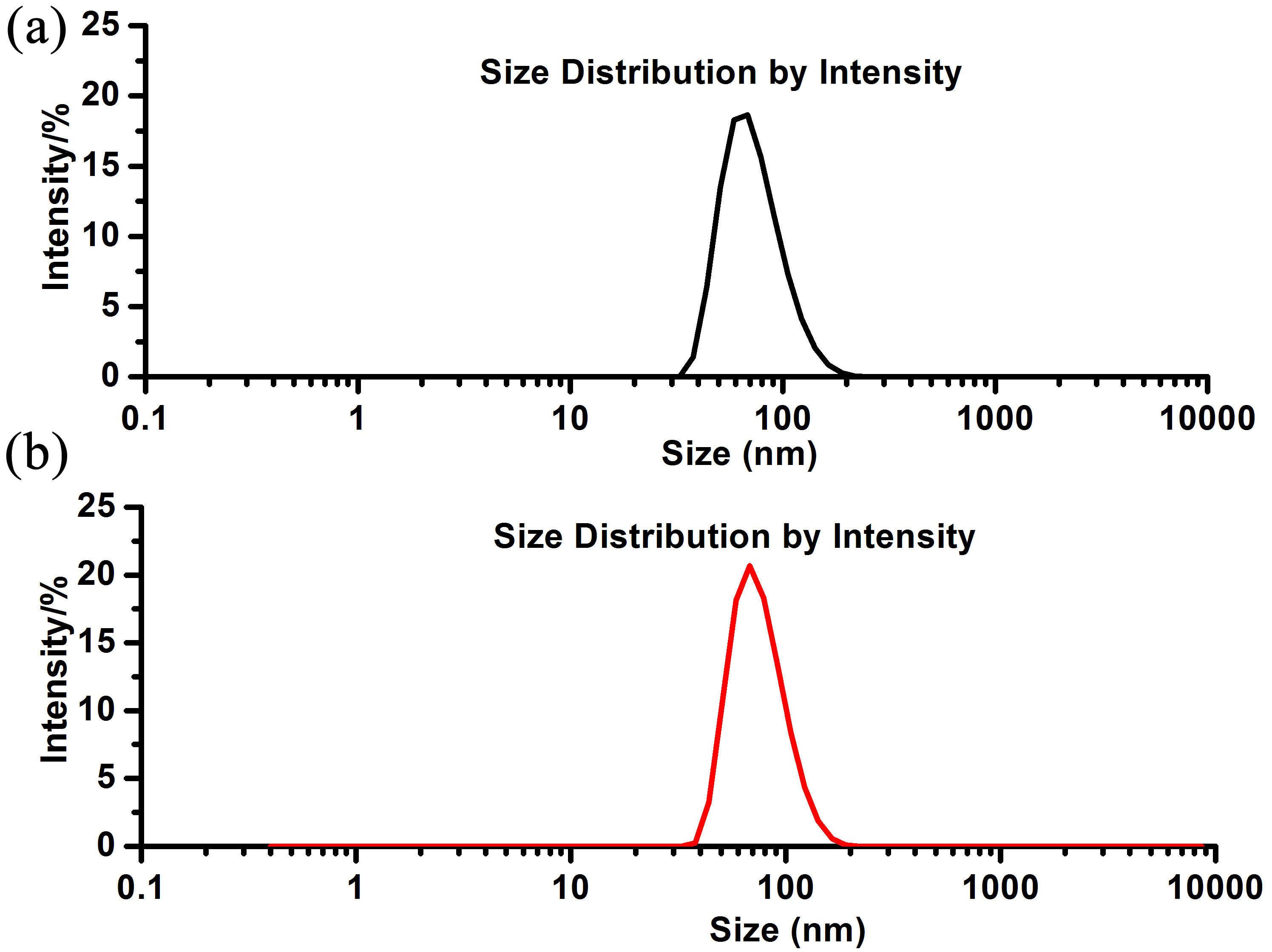


Figure S1. Diameter distribution of (a) AuNSs@PDA NPs and (b) AuNSs@PDA-RGD NPs.


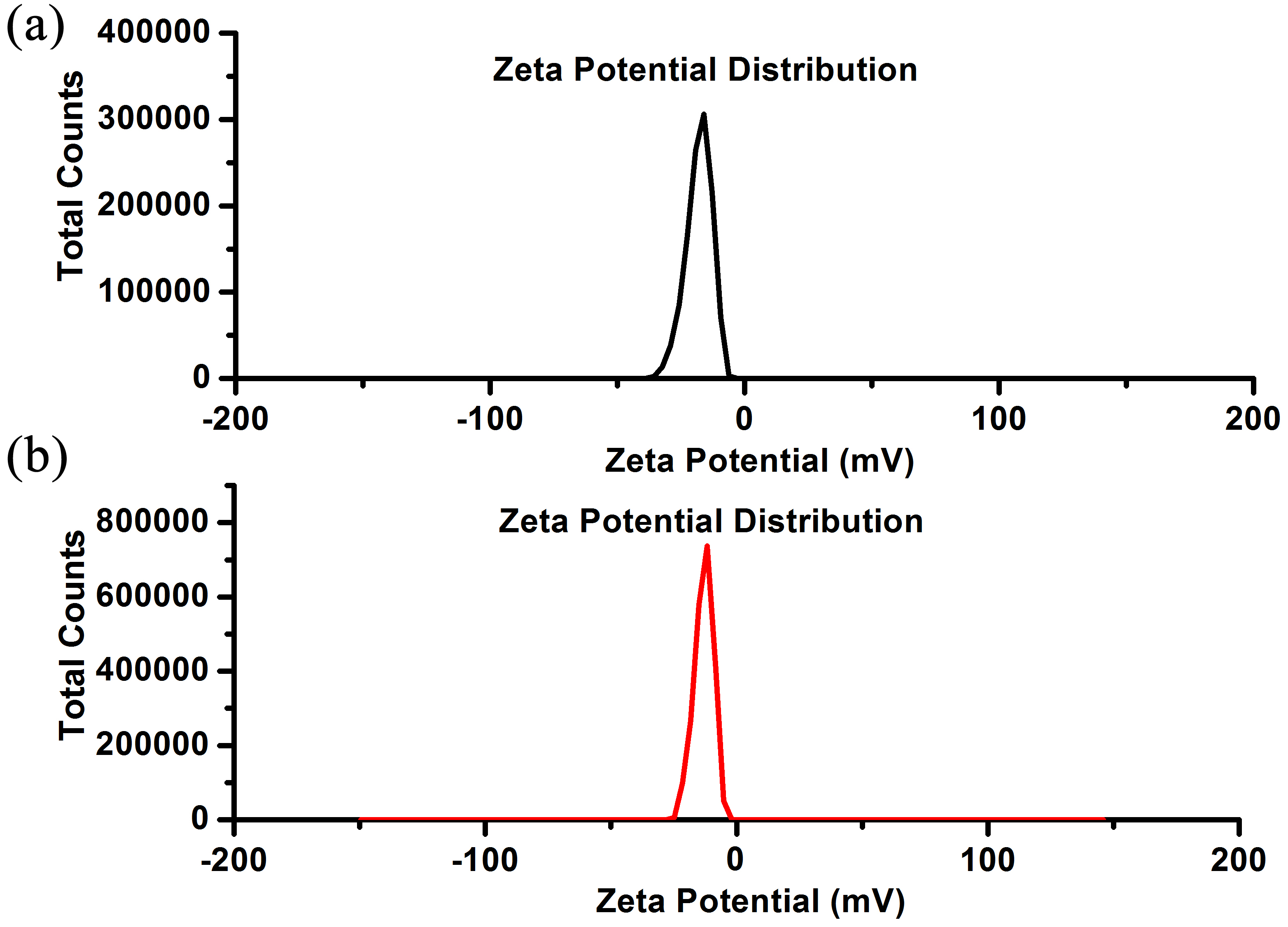


Figure S2. Zeta potential of (a) AuNSs@PDA NPs and (b) AuNSs@PDA-RGD NPs.


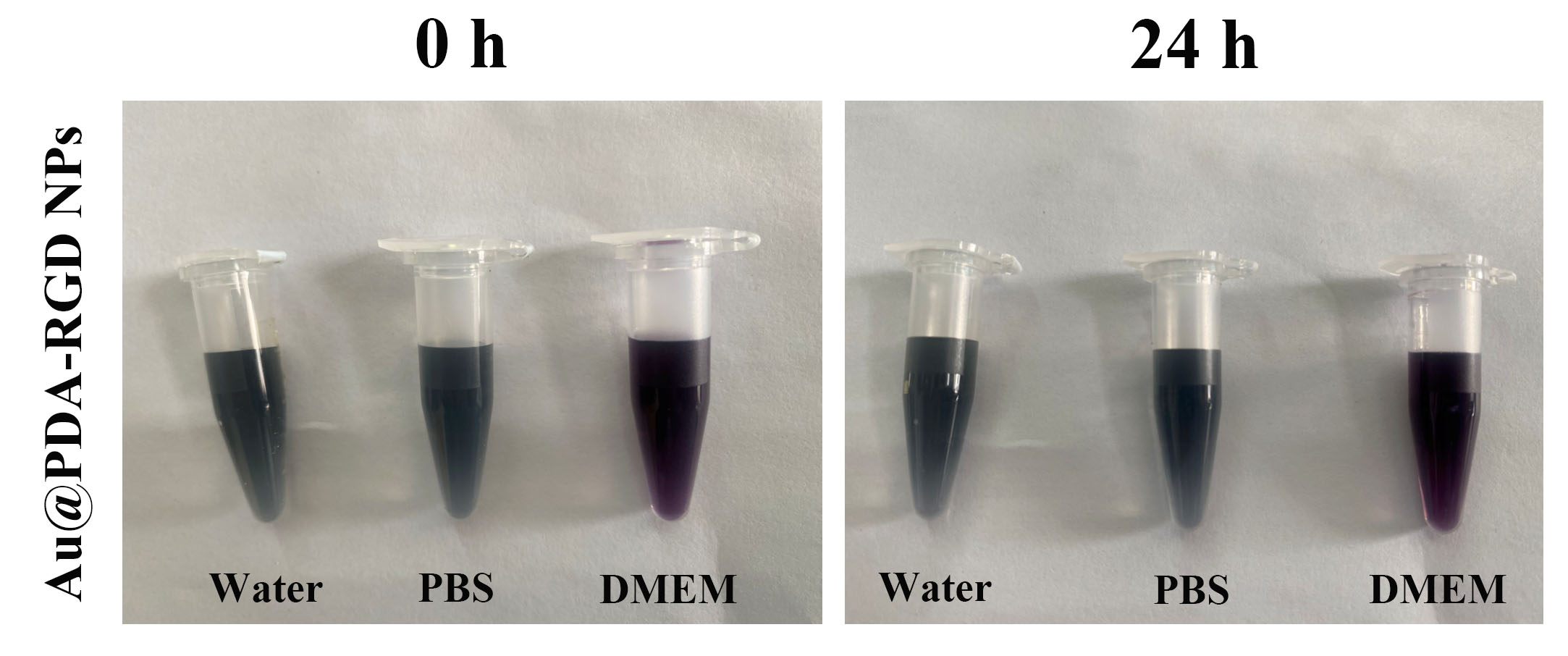


Figure S3. Digital photographs of AuNSs@PDA-RGD NPs in three different dispersions at 0 and 24 h.


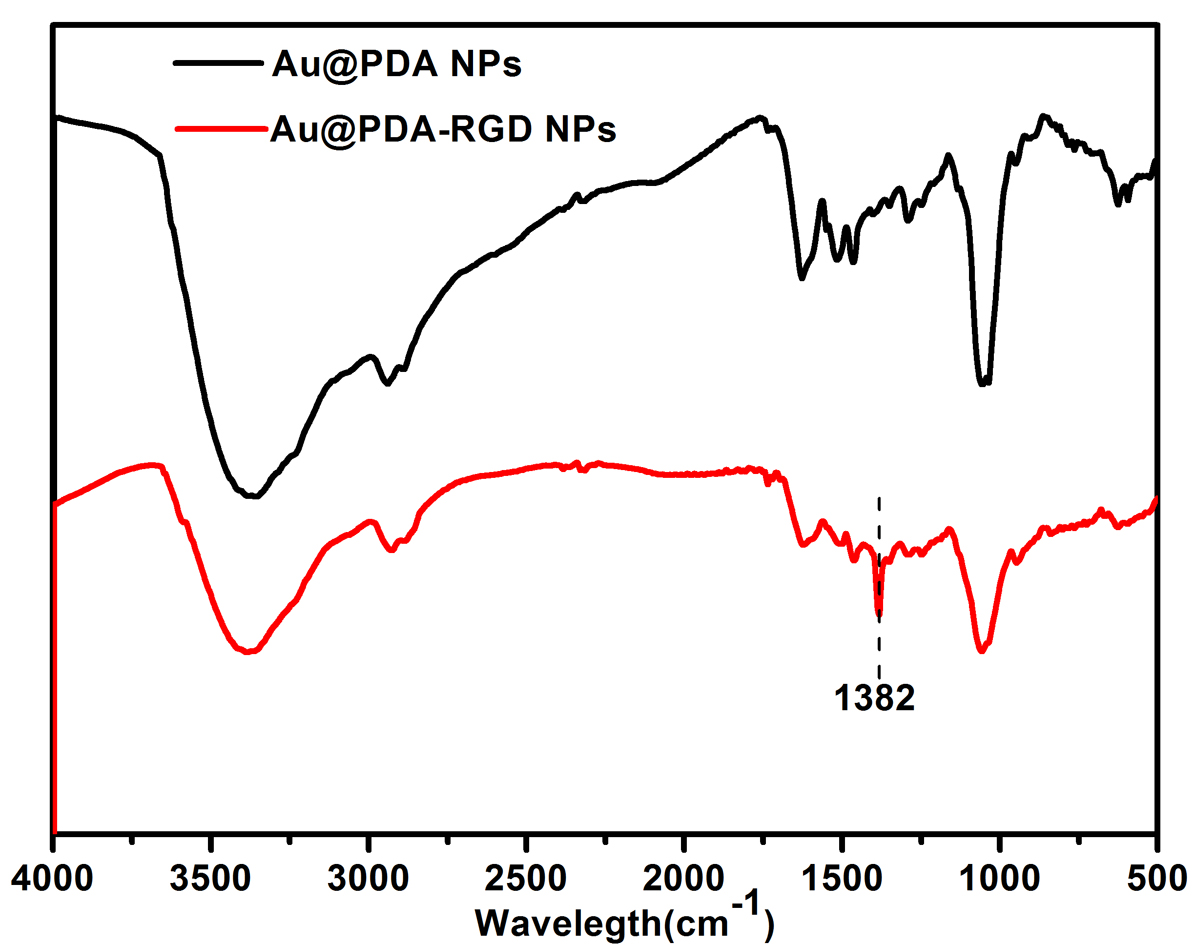


Figure S4. FTIR spectra of AuNSs@PDA NPs and AuNSs@PDA-RGD NPs.


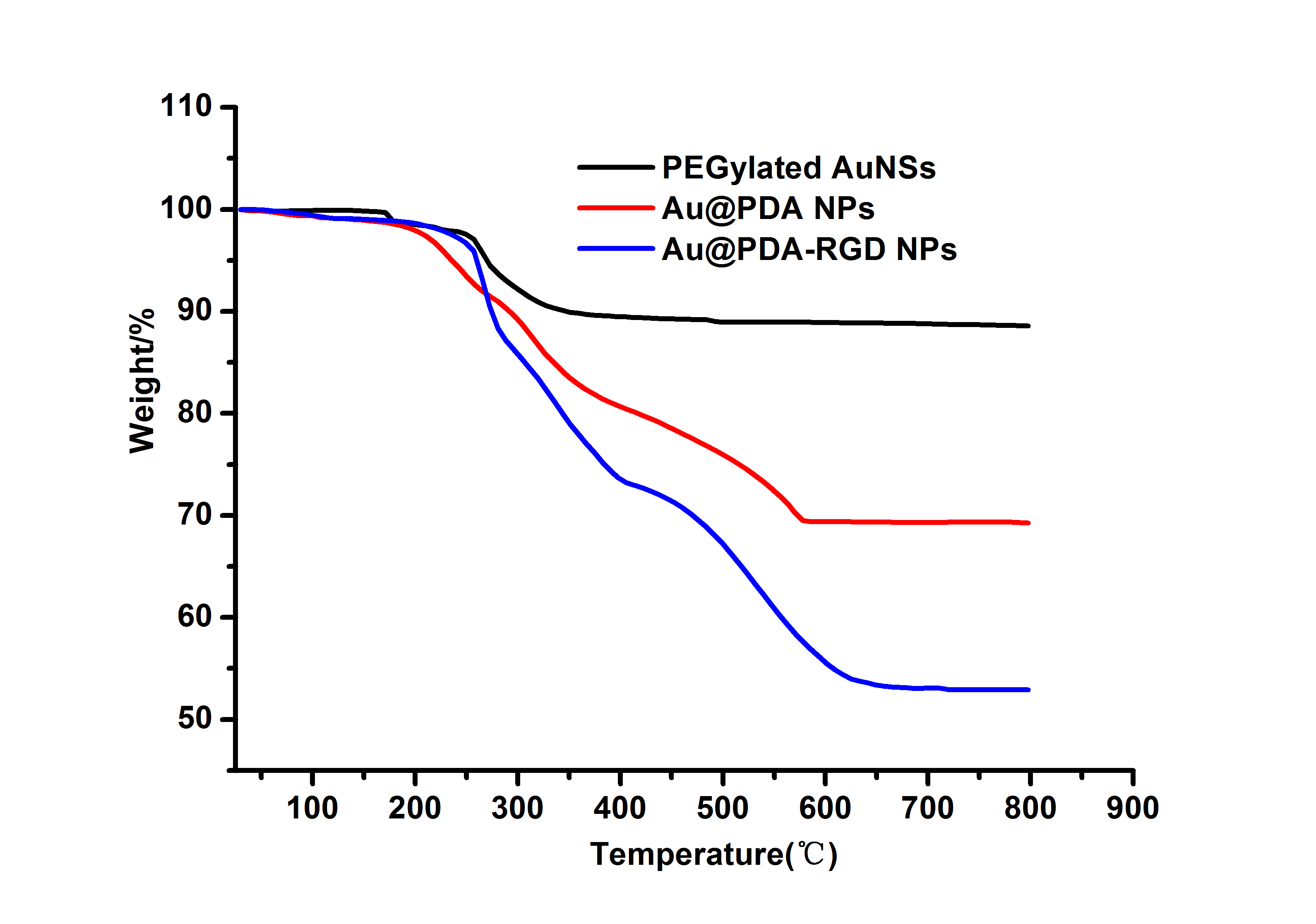


Figure S5. TGA curves of PEGylated AuNSs, Au@PDA NPs and Au@PDA-RGD NPs.


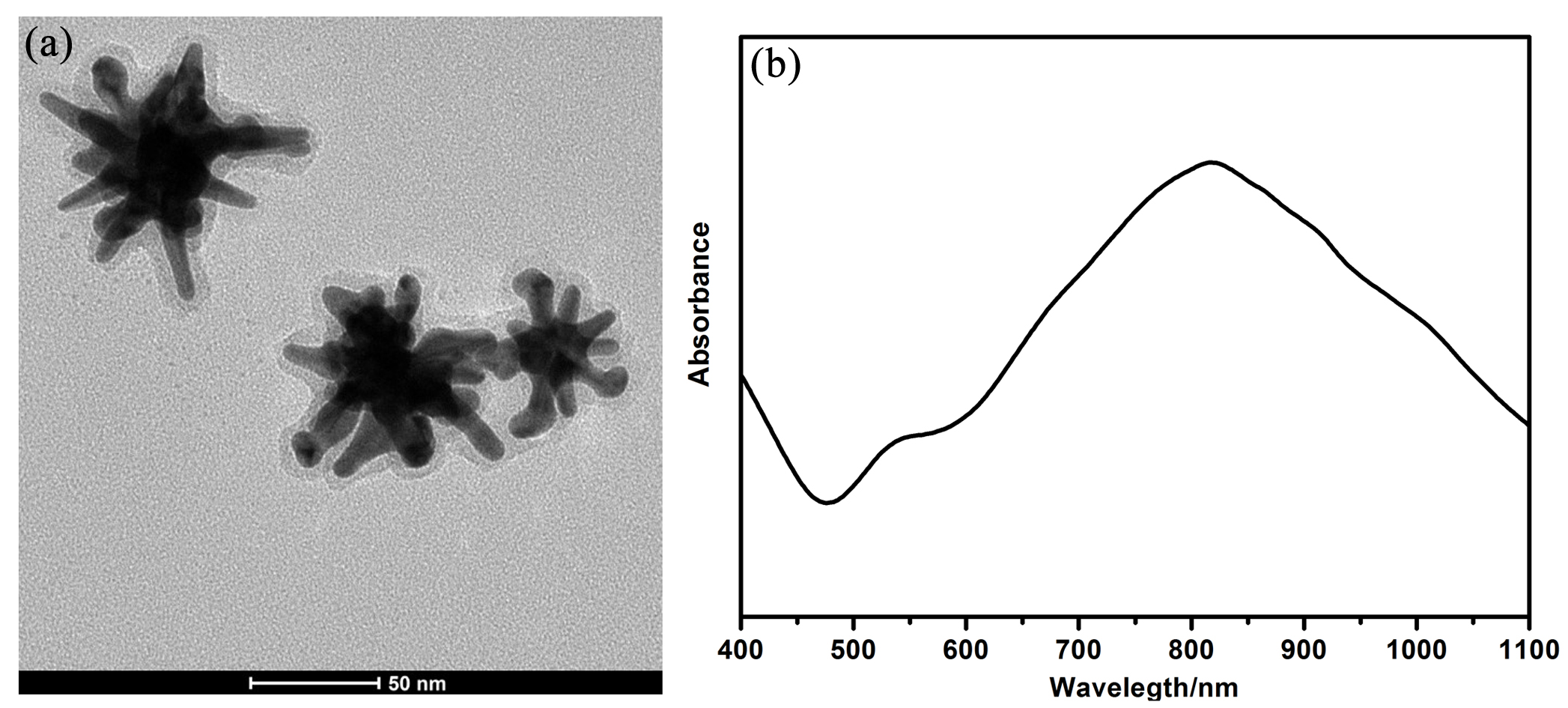


Figure S6. (a) TEM image and (b) UV-vis-NIR spectrum of AuNSs@PDA-RGD NPs after laser irradiation.


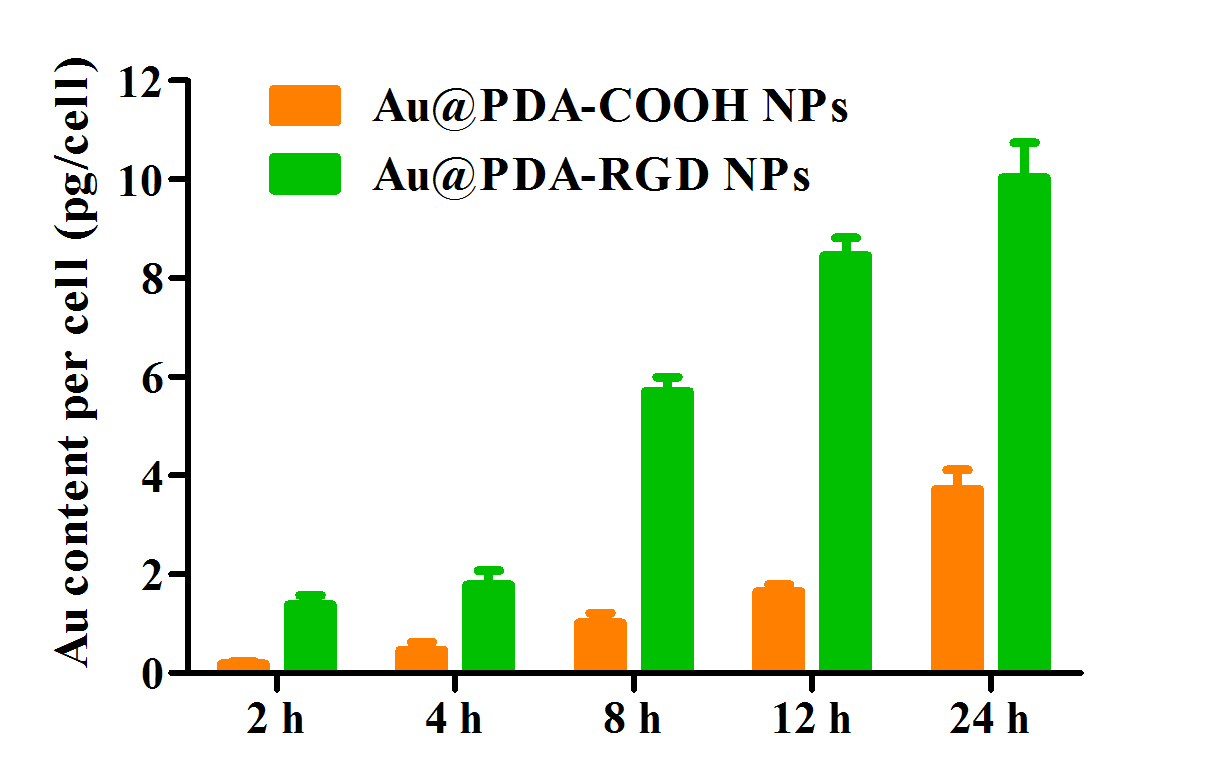


Figure S7. Au contents in HepG2 cells after incubation with Au@PDA-COOH NPs or Au@PDA-RGD NPs for different periods of time.


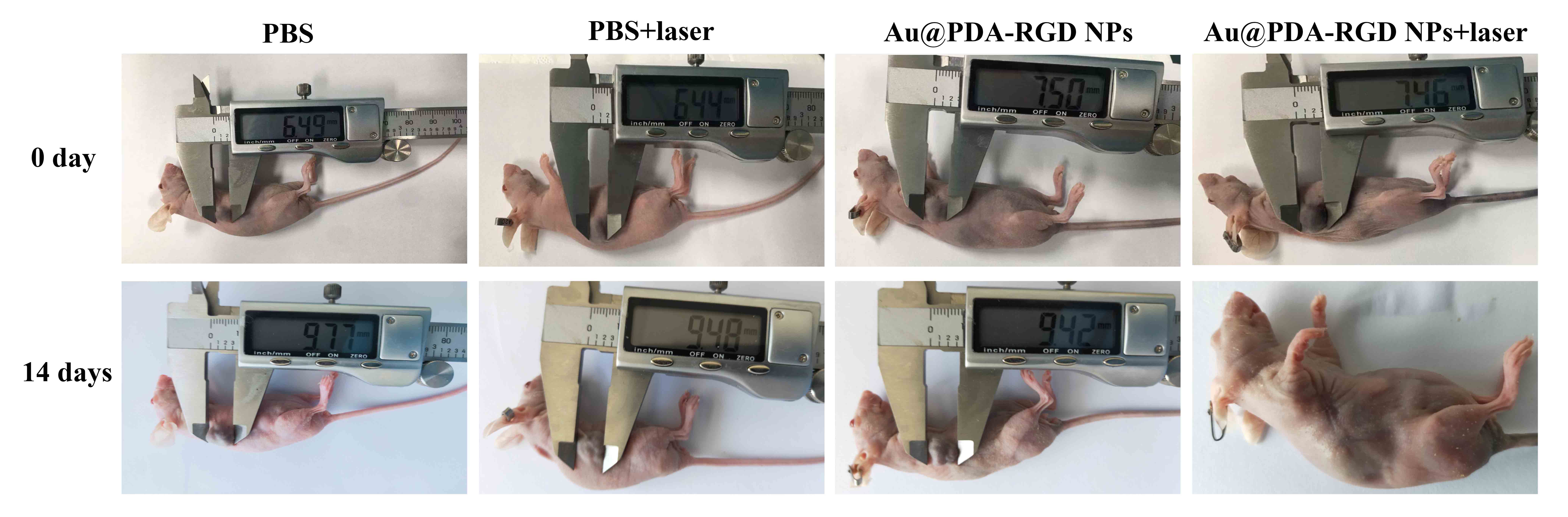


Figure S8. Representative pictures for mice bearing HepG2 tumor at the 0th and 14th day of treatment.


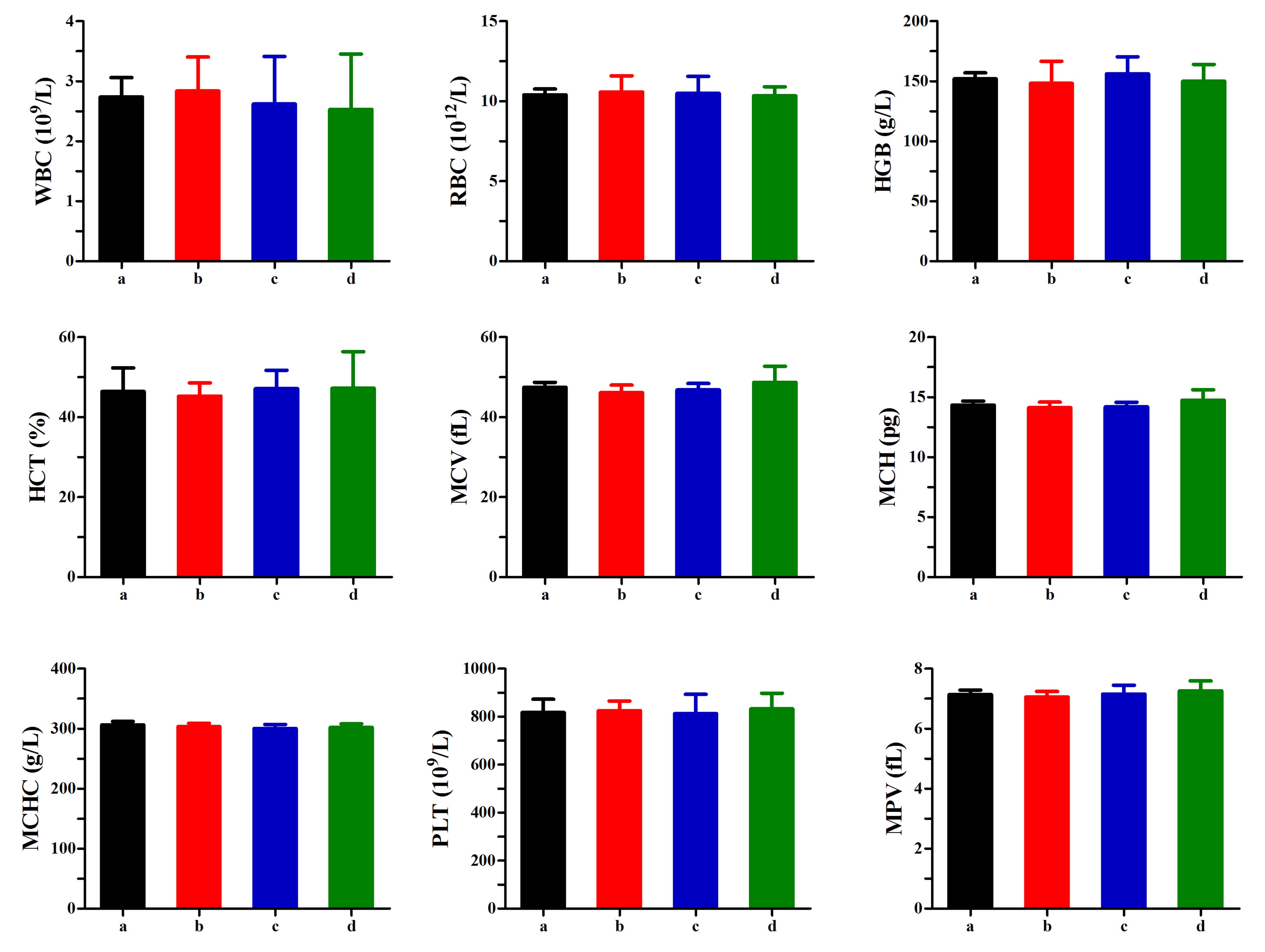


Figure S9. Blood routine examination of mice after different treatments. a, PBS; b, PBS + laser; c, Au@PDA-RGD NPs; d, Au@PDA-RGD NPs + laser. Data were presented as mean ± SD (n = 5). White Blood Cell (WBC), Red Blood Cell (RBC), Hemoglobin (HGB), Red Blood Cell Specific Volume (HCT), Erythrocyte Mean Corpuscular Volume (MCV), Mean Corpuscular Hemoglobin (MCH), MCH/MCV (MCHC), Platelet (PLT), Mean Platelet Volume (MPV).


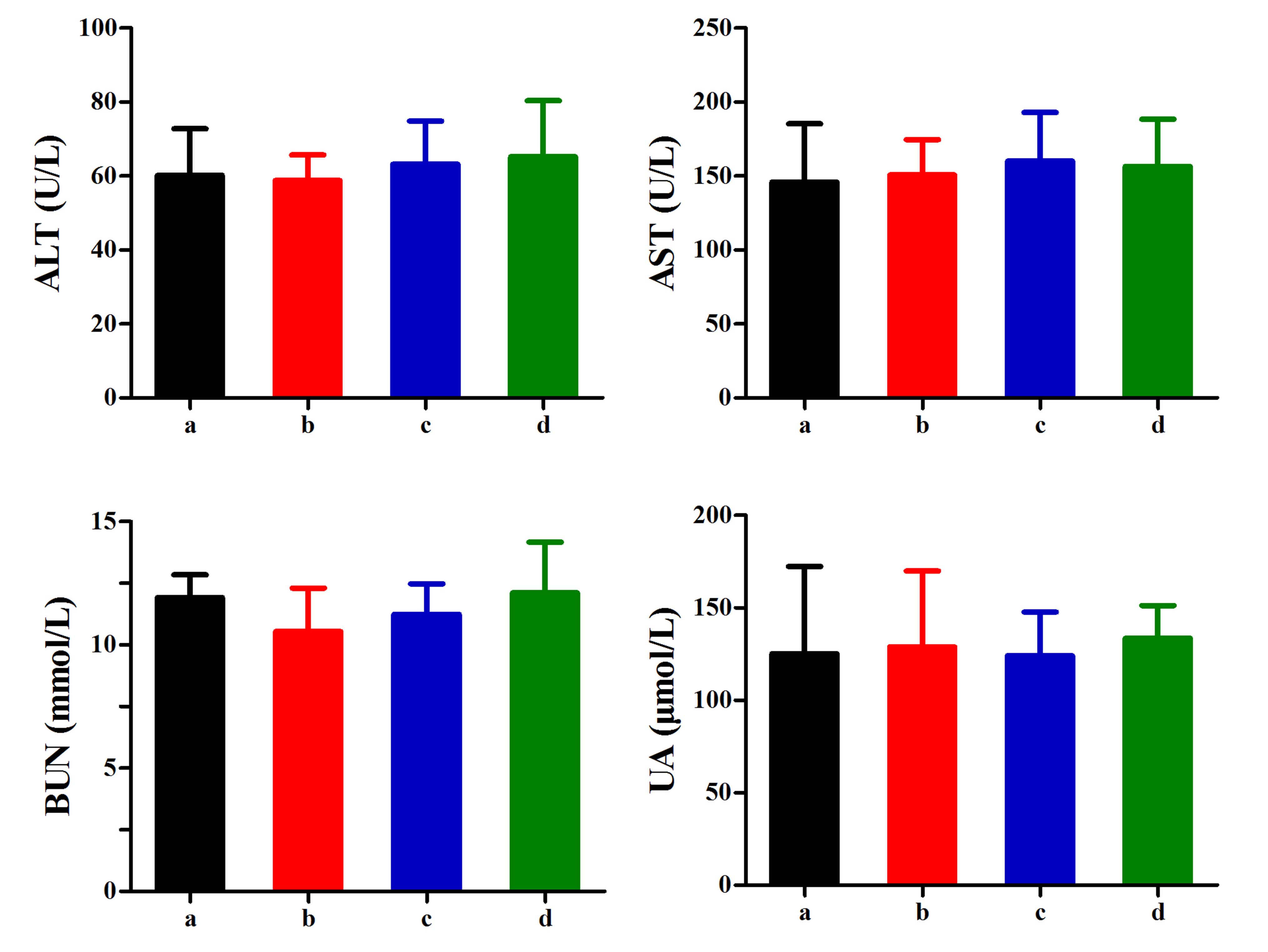


Figure S10. Blood biochemistry analysis of mice after different treatments. a, PBS; b, PBS + laser; c, Au@PDA-RGD NPs; d, Au@PDA-RGD NPs + laser. Data were presented as mean ± SD (n = 5). Alanine Aminotransferase (ALT), Aspartate Aminotransferase (AST), Blood Urea Nitrogen (BUN), Uric Acid (UA).
